# Acute obinutuzumab-induced thrombocytopenia in follicular lymphoma: case report and literature review

**DOI:** 10.3389/fonc.2025.1643313

**Published:** 2025-10-17

**Authors:** Jieyi Zhou, Miaomiao Chen, Yi Zhang, Ce Xu, Rong Li

**Affiliations:** ^1^ Department of Nuclear Radiation Injury Protection and Treatment, Naval Medical Center, Naval Medical University, Shanghai, China; ^2^ The Department of Hematology, Navy Medical Center of People's Liberation Army, Shanghai, China

**Keywords:** obinutuzumab, thrombocytopenia, immune mechanism, follicular lymphoma, hematologic malignancies, case report

## Abstract

**Introduction:**

Obinutuzumab, a type II anti-CD20 monoclonal antibody used in the treatment of follicular lymphoma, is associated with a higher incidence of adverse events, including thrombocytopenia, compared to rituximab. We report a case of severe, acute thrombocytopenia induced by obinutuzumab and explore its potential mechanism.

**Case presentation:**

A 47-year-old woman with follicular lymphoma developed recurrent, severe (grade III–IV) thrombocytopenia within 24 h following obinutuzumab administration, starting from her second treatment cycle. Subsequent bone marrow aspiration revealed significant megakaryocyte hyperplasia but with impaired platelet production. Key laboratory findings included elevated levels of pro-inflammatory cytokines TNF-α and IL-6, decreased complement levels, and reduced IgM, while the platelet antibodies tested were negative. The patient’s thrombocytopenia was managed and improved with treatments including recombinant human thrombopoietin (rhTPO) and platelet transfusions.

**Conclusion:**

Obinutuzumab can rarely cause acute, severe thrombocytopenia, possibly through a combination of immune-mediated platelet destruction and impaired megakaryocyte maturation.

## Background

Obinutuzumab is a humanized, glycosylated type II anti-CD20 monoclonal antibody (IgG1/κ). Compared with type I monoclonal antibody such as rituximab, it exhibits significantly different effects of antibody-dependent cell-mediated cytotoxicity (ADCC) and direct cell death (DCD), while its capacity to induce complement activation is milder (1, 2). Additionally, it has been reported to have a higher incidence of adverse events.

Herein we report a case of a 47-year-old woman diagnosed with follicular lymphoma (FL) who developed obinutuzumab-induced acute thrombocytopenia (OIAT). Through bone marrow aspiration performed before and after treatment, we further investigated the underlying mechanism.

## Case report

The patient was diagnosed with FL (low-grade, Ann Arbor stage IV) at the age of 47. PET-CT revealed lymphadenopathy and splenomegaly. Initially, there was no indication for treatment. After 5 months of observation, she began complaining of abdominal distention and discomfort. Abdominal imaging confirmed a high tumor burden necessitating treatment. Bone marrow aspiration and trephine biopsy showed bone marrow infiltration by FL. The patient was started on treatment with obinutuzumab (1,000 mg, d0) and bendamustine (150 mg, d1-2, 90 mg/m^2^) at another hospital. After a three-cycle treatment, she reached complete response (CR). Following the first course, the platelet nadir was 199 × 10^9^/L. After the second course, the platelet nadir was 44 × 10^9^/L, and recombinant human thrombopoietin (rhTPO) was administered once. Platelet recovery time was 16 days. The minimum platelet count in the third course of treatment is 7 × 10^9^/L, and rhTPO was administered four times. In the fourth course, the platelet nadir was 4 × 10^9^/L, requiring rhTPO administration seven times and two units of apheresis platelets. The time for platelet recovery to 80 × 10^9^/L was 20 days. The patient was then admitted to our hospital for the fifth course of treatment.

**Figure 1:**
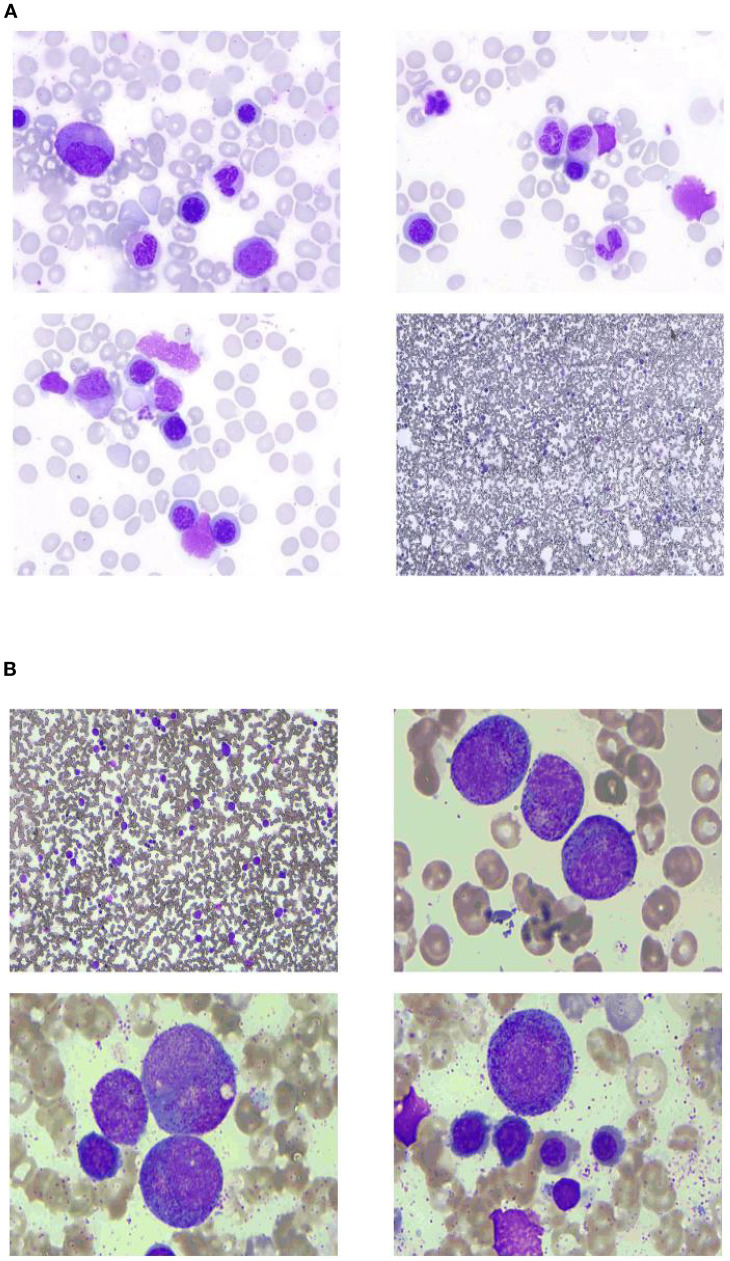
Bone marrow smear **(A)** before and **(B)** after the fifth course.

**Figure 2:**
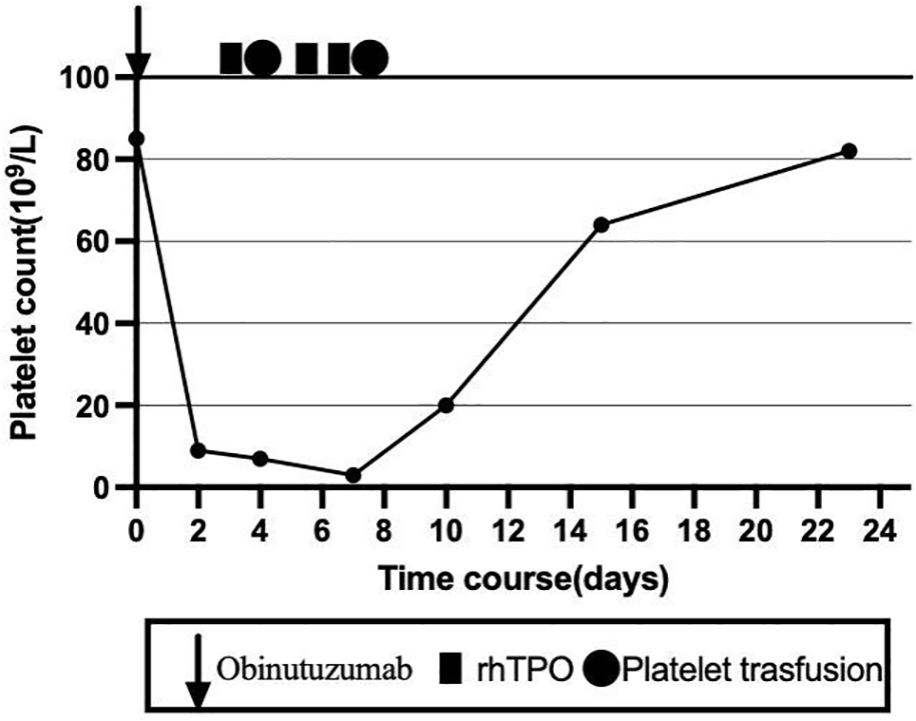
Changes of platelet count and treatment overview during the fifth course.

To clarify the cause of thrombocytopenia, laboratory tests were performed to exclude comorbidities (such as chronic viral hepatitis and autoimmune diseases) that could influence immune-mediated thrombocytopenia. Bone marrow aspiration was performed at the left posterior superior iliac spine before and after the fifth course (**Figure 1**). Before treatment, there were 78 megakaryocytes, 52 (44–60) granulosa megakaryocytes, 36 (28–48) platelet-producing megakaryocytes, and eight naked megakaryocytes (8), with adequate platelet production and scattered platelets observed. After treatment, there were 215 megakaryocytes, including 92 granulocytic megakaryocytes (44–60), four platelet-producing megakaryocytes (28–48), and four naked megakaryocytes, with impaired platelet production and rare platelets. Laboratory tests on the second day after the GB regimen showed the following results: WBC 4.8 × 10^9^/L, platelets 9 × 10^9^/L, normal coagulation function, negative platelet antibodies, elevated tumor necrosis factor-α (TNF-α), elevated interleukin-6 (IL-6), and significantly decreased complement levels. The patient received two units of apheresis platelet, subcutaneous rhTPO injections three times, and symptomatic treatment. The platelet nadir was 3 × 10^9^/L at C5D7. The platelets recovered to 20 × 10^9^/L at C5D10 and 80 × 10^9^/L after 23 days (**Figure 2**).

For the sixth course, we advised switching to bendamustine and rituximab (BR) regimen. However, after thorough communication, the patient requested to continue the GB regimen. Given her treatment response, absence of significant bleeding manifestations, and acceptable platelet recovery, we ultimately complied with her request.

**Figure 3:**
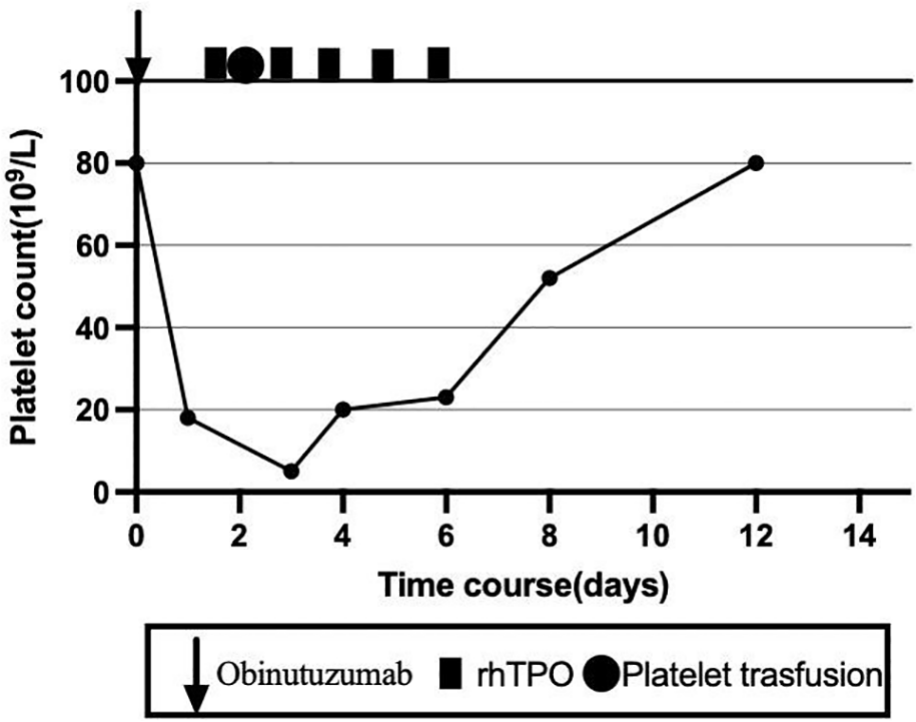
Changes of platelet count and treatment overview during the sixth course.

The patient subsequently completed the sixth cycle of the GB regimen. Following obinutuzumab infusion on C6D1, the platelet count dropped from 80 × 10^9^/L to 18 × 10^9^/L within 1 day. To reduce the bleeding risk, the patient was advised to strictly limit her activity. Meanwhile, rhTPO was administered five times, and herombopag (5 mg once daily) was given for 7 days. One unit of apheresis platelets was transfused on C6D2. The platelets recovered to 20 × 10^9^/L on C6D4 and to 80 × 10^9^/L on C6D12 (**Figure 3**). After six cycles of GB therapy, the patient achieved CR as evaluated by PET-CT. She was then scheduled to receive two additional cycles of rituximab and maintenance therapy with rituximab every 2 months.

## Discussion

Literature reports indicate that the overall risk of severe infusion-related reactions (IRR) and thrombocytopenia (≥ grade 3) with obinutuzumab is at least twice as high as with rituximab (3). IRR induced by both rituximab and obinutuzumab has been attributed to cytokine release. Studies have found that patients experiencing IRRs release more IL-8, IL-6, and TNF-α than those without IRRs. This phenomenon appears to be limited to the first infusion and is accompanied by the rapid destruction of circulating B cells and a reduction in NK cell numbers (4). Similarly, severe thrombocytopenia is most common during the first cycle and is associated with risk factors such as high tumor burden, high CD20 expression, splenomegaly, and bone marrow infiltration (5). Obinutuzumab appears to induce a stronger cytokine release syndrome, leading to higher rates of IRRs and thrombocytopenia (6).

This patient did not exhibit significant thrombocytopenia after the first treatment but developed grade III thrombocytopenia in the second course and grade IV in the subsequent courses. Follow-up blood tests revealed that thrombocytopenia occurred within 24 h after obinutuzumab administration. The bone marrow aspirates before and after treatment showed significant megakaryocyte hyperplasia with impaired platelet production, and the platelet antibody was negative. The cytokine levels (TNF-α and IL-6) were elevated, IgM was continuous, and the complement levels were reduced. Obinutuzumab has a weaker complement-dependent cytotoxicity (CDC) effect but is more commonly associated with cytokine storm or the rapid destruction of circulating B cells and NK cells, leading to thrombocytopenia. Bone marrow examination revealed acute impairment of megakaryocyte maturation, a feature not commonly reported in cases of acute thrombocytopenia. Some cases of rituximab-induced acute thrombocytopenia have been reported, often related to tumor burden and bone marrow infiltration. However, our patient continued to experience thrombocytopenia after achieving bone marrow remission, suggesting that acute thrombocytopenia induced by obinutuzumab may be an underrecognized and rare adverse event.

To date, nearly 20 cases of OIAT have been reported in the literature. A PubMed search using the terms “obinutuzumab” and “acute thrombocytopenia” identified eight cases in FL. **Table 1** summarizes the basic information of these cases. Sakai et al. (2020) reported a case of thrombocytopenia in a patient with relapsed FL treated with obinutuzumab combined with bendamustine, occurring from the first induction therapy and persisting during maintenance therapy. Thrombocytopenia began 1 h after infusion, reached its nadir after 4 days, and required more than 10 days for recovery (7). Haage et al. (2022) reported a previously untreated FL patient with high tumor burden and bone marrow involvement who developed grade IV thrombocytopenia after the first obinutuzumab administration and was treated with intravenous immunoglobulin, recovering within 4 days (8). In the GALLIUM trial, cytopenia occurred in approximately 11.4% of obinutuzumab-treated patients, with 6.1% being ≥grade III (9). Mechelfekh et al. reported a 74-year-old woman with FL whose platelets dropped from 376 × 10^9^/L to 3 × 10^9^/L after obinutuzumab-CVP treatment (10).

**Table 1:** Summary of all cases reported in the literature and the current OIAT case.

Reference	Disease	Course	Treatment plan	Pre-treatment platelet count (10^9^/L)	Platelet nadir after treatment (10^9^/L)	Management	Outcome	Rechallenge with anti-CD20 (recurrence)
Walter et al. (1)	CLL	1	G+Ven	150	40	Platelet transfusion	+7d recovery to grade 1	Yes (no)
Sakai et al. (7)	FL	1	G	160	21	Platelet transfusion	+10d recovery to normal	Yes (yes)
Haage et al. (8)	FL	1	G+B	245	4	Platelet transfusion, IVIG	+6d recovery to normal	Switched to R (no)
Mechelfekh et al. (10)	FL	1	G+CVP	376	3	Platelet transfusion, romiplostim	+22d recovery to normal	Switched to R (no)
Mechelfekh et al. (10)	MCL	1	G+Glo	76	3	Platelet transfusion	+21d recovery to grade 1	Yes (yes)
Ng, J.Y. et al. (13)	CLL	1	G+Ven	104	4	Platelet transfusion	+8d recovery to normal	Yes (yes)
Yilmaz et al. (14)	DLBCL	1	G+Len	144	33	Platelet transfusion	+5d recovery to grade 1	Yes (no)
Yilmaz et al. (14)	FL	1	G+ICE	111	23	Platelet transfusion	+4d recovery to grade 2	Yes (no)
Yilmaz et al. (14)	FL	1	G+ICE	112	13	Platelet transfusion	+23d still dependent on transfusion	Yes (no)
Dou X et al. (15)	FL	1	G+B	130	27	Clinical observation	+8d recovery to normal	Yes (yes)
Dou X et al. (15)	FL	2	G+B	192	15	Platelet transfusion	+29d recovery to grade1	Switched to R (no)
Tane M et al. (16)	FL	1	G+B	222	11	Platelet transfusion	+29d recovery to normal	Switched to R (no)
Current case (fifth course)	FL	5	G+B	85	3	Platelet transfusion, rhTPO*3d	+23d recovery to grade 1	No (yes)
Current case (sixth course)	FL	6	G+B	80	5	rhTPO*5d, Hetrombopag*7d	+16d recovery to grade 1	Switched to R (no)

NR, not reported.

OIAT has also been observed in other diseases. Freeman et al. reported that in some patients with chronic lymphocytic leukemia, the first administration of obinutuzumab directly released proinflammatory cytokines IL-6 and IL-8, and immune-mediated platelet lysis via CD20 antigen presentation or complement activation by circulating soluble CD20 antigen resulted in acute thrombocytopenia (5). An analysis of this patient’s thrombocytopenia suggested an immune-related mechanism.

Compared to obinutuzumab, there are more reported cases and studies on rituximab-induced acute thrombocytopenia (RIAT). Generally accepted risk factors for RIAT include bone marrow involvement, splenomegaly, infusion-related reactions (cytokine release syndrome), and mantle cell lymphoma (MCL). The mechanism of RIAT remains unclear. Possible mechanisms include the following: Qiao et al. proposed that platelets express FcγRIIa (CD32a), which binds to IgG immune complexes and mediates platelet degradation when lymphoma-cell-bound rituximab interacts with platelets via Fc receptor (11). Qureini et al. suggested that rituximab interacts with tumor cells, activates the immune system, induces consumptive coagulopathy and hyperfibrinolysis, and reduces platelet counts. Additionally, rituximab may form immune complexes that activate complement, leading to platelet destruction, or promote cytokine release including TNF-α (12). Robinson et al. demonstrated that rituximab infusion induces the premature formation of anti-CD20 immune complexes and immune-mediated cell lysis (17). Depletion of lymphoma cells by rituximab may expose the endothelium, impair the endothelial barrier, and induce platelet activation and aggregation.

This patient had splenomegaly and bone marrow involvement at the onset. After treatment, PET-CT showed CR, but splenomegaly persisted, and the IgM levels remained below normal. We hypothesize that obinutuzumab likely induced immune complex formation, direct release of proinflammatory factors (IL-6 and IL-8), immune-mediated platelet lysis via CD20 antigen presentation, complement activation, IgM depletion, and endothelial exposure post-lymphoma cell clearance, leading to platelet activation, aggregation, and destruction. This may explain why thrombocytopenia was not apparent after the first treatment but worsened with subsequent cycles. Combined bone marrow suppression from bendamustine may also have contributed. Recently, Kars et al. reported a case of severe RIAT in splenic marginal zone lymphoma, further highlighting the immune-mediated nature of this adverse event (18). However, our case uniquely demonstrates acute megakaryocyte maturation dysfunction, a feature not emphasized in most RIAT reports, suggesting a potentially distinct pathophysiology for obinutuzumab.

## Conclusion

Acute thrombocytopenia induced by obinutuzumab is a rare but serious adverse reaction with rapid onset, significantly impacting patient safety. Currently, few cases have been reported, and the mechanism remains unclear. In this patient, we observed significant megakaryocyte hyperplasia with impaired platelet production before and after obinutuzumab treatment. The clinical presentation differed from previously reported cases, suggesting that the mechanism of obinutuzumab-induced acute thrombocytopenia may involve multiple pathways, warranting further investigation.

## Data Availability

The original contributions presented in the study are included in the article/supplementary material. Further inquiries can be directed to the corresponding author.

## References

[1] Walter HS Jayne S Mensah P Miall FM Lyttelton M Dyer MJ . Obinutuzumab-induced coagulopathy in chronic lymphocytic leukaemia with trisomy 12. Blood Cancer J. (2016) 6:e435. doi: 10.1038/bcj.2016.42, PMID: 27315112 PMC5141357

[2] Mossner E Brunker P Moser S Puntener U Schmidt C Herter S . Increasing the efficacy of CD20 antibody therapy through the engineering of a new type II anti-CD20 antibody with enhanced direct and immune effector cell-mediated B-cell cytotoxicity. Blood. (2010) 115:4393–402. doi: 10.1182/blood-2009-06-225979, PMID: 20194898 PMC2881503

[3] Hong X Song Y Shi Y Zhang Q Guo W Wu G . Efficacy and safety of obinutuzumab for the first-line treatment of follicular lymphoma: a subgroup analysis of Chinese patients enrolled in the phase III GALLIUM study. Chin Med J (Engl). (2021) 135:433–40. doi: 10.1097/CM9.0000000000001737, PMID: 35194005 PMC8869628

[4] Freeman CL Dixon M Houghton R Kreuzer KA Fingerle-Rowson G Herling M . Role of CD20 expression and other pre-treatment risk factors in the development of infusion-related reactions in patients with CLL treated with obinutuzumab. Leukemia. (2016) 30:1763–6. doi: 10.1038/leu.2016.41, PMID: 26979130 PMC4980557

[5] Freeman CL Morschhauser F Sehn L Dixon M Houghton R Lamy T . Cytokine release in patients with CLL treated with obinutuzumab and possible relationship with infusion-related reactions. Blood. (2015) 126:2646–9. doi: 10.1182/blood-2015-09-670802, PMID: 26447188 PMC4671111

[6] Freeman CL Sehn LH . A tale of two antibodies: obinutuzumab versus rituximab. Br J Haematol. (2018) 182:29–45. doi: 10.1111/bjh.15232, PMID: 29741753

[7] Sakai K Matsumura T Hamada R Tominaga T Takahashi T . Acute thrombocytopenia after obinutuzumab administration in a patient with relapsed follicular lymphoma. Rinsho Ketsueki. (2020) 61:1616–9. doi: 10.11406/rinketsu.61.1616, PMID: 33298656

[8] Haage TR Surov A Mougiakakos D Berisha M . Successful use of intravenous immunoglobulins in an obinutuzumab-related acute thrombocytopenia. Hemasphere. (2022) 6:e751. doi: 10.1097/HS9.0000000000000751, PMID: 35935607 PMC9351898

[9] Hiddemann W Barbui AM Canales MA Cannell PK Collins GP Durig J . Immunochemotherapy with obinutuzumab or rituximab for previously untreated follicular lymphoma in the GALLIUM study: influence of chemotherapy on efficacy and safety. J Clin Oncol. (2018) 36:2395–404. doi: 10.1200/JCO.2017.76.8960, PMID: 29856692

[10] Mechelfekh Y Pontrucher A Paillassa J Temple M Houot R . Obinutuzumab-induced acute thrombocytopenia: Report of two cases and review of literature. Br J Haematol. (2023) 202:168–72. doi: 10.1111/bjh.18826, PMID: 37143375

[11] Qiao J Al-Tamimi M Baker RI Andrews RK Gardiner EE . The platelet Fc receptor, FcgammaRIIa. Immunol Rev. (2015) 268:241–52. doi: 10.1111/imr.12370, PMID: 26497525

[12] Qureini A Asif S Harry S Madhusudhana S . A case of rituximab-induced acute thrombocytopenia in a patient with splenic marginal zone lymphoma and chronic hepatitis C virus infection. Am J Case Rep. (2019) 20:1394–7. doi: 10.12659/AJCR.917644, PMID: 31541071 PMC6767946

[13] Ng JY Joshi M Choi P . Frequency and outcomes of obinutuzumab-induced thrombocytopenia. Br J Haematol. (2023) 203:668–72. doi: 10.1111/bjh.19147, PMID: 37853574

[14] Yilmaz U Kucukyurt S Ar MC Eskazan AE . Acute thrombocytopenia complicating the initial administration of obinutuzumab: is it more frequent than we think? Oncol Ther. (2024) 12:157–61. doi: 10.1007/s40487-023-00259-y, PMID: 38252230 PMC10881447

[15] Dou X Li K Lu J . Obinutuzumab-induced acute thrombocytopenia: a case report and literature review. Front Oncol. (2024) 14:1509567. doi: 10.3389/fonc.2024.1509567, PMID: 39741978 PMC11685111

[16] Tane M Hosoi H Hiroi T Murata S Mushino T Sonoki T . Obinutuzumab-induced acute thrombocytopenia mimicking immune thrombocytopenia in a patient with follicular lymphoma. Intern Med. (2025). doi: 10.2169/internalmedicine.5191-24, PMID: 40222942 PMC12589149

[17] Robinson AC Nachar VR . Successful rechallenge of rituximab following severe rituximab-induced acute thrombocytopenia in a patient with splenic marginal zone lymphoma. J Oncol Pharm Pract. (2020) 26:1248–53. doi: 10.1177/1078155219890023, PMID: 31766968

[18] Kars TU Yorganci ZF Yaskiran O Tekinalp A Demircioglu S . Rituximab-induced severe acute thrombocytopenia in a patient with splenic marginal zone lymphoma. J Oncol Pharm Pract. (2023) 29:1011–4. doi: 10.1177/10781552221142870, PMID: 36458320

